# Supplementary material for: Construction of an Alternative NAD+ De Novo Biosynthesis Pathway
Source: Adv Sci (Weinh). 2021 Mar 1;8(9):2004632. doi: 10.1002/advs.202004632 (PMC8097395; doi:10.1002/advs.202004632)
Supplement: Supplementary file 1 — Supporting Information [file ADVS-8-2004632-s001.pdf]

WILEY-VCH

© 2021 Wiley-VCH GmbH

ADVANCED SCIENCE
Open Access

Supporting Information

for *Adv. Sci.*, DOI: 10.1002/advs.202004632

Construction of an alternative NAD^+ *de novo* biosynthesis pathway

*Yong Ding, Xinli Li, Geoff P. Horsman, Pengwei Li, Min Wang, Jine Li, Zhilong Zhang, Weifeng Liu, Bian Wu, Yong Tao, and Yihua Chen**

Supporting Information

Construction of an alternative NAD^+ *de novo* biosynthesis pathway

*Yong Ding, Xinli Li, Geoff P. Horsman, Pengwei Li, Min Wang, Jine Li, Zhilong Zhang, Weifeng Liu, Bian Wu, Yong Tao, and Yihua Chen**

Y. Ding, X. Li, Dr. P. Li, M. Wang, Dr. J. Li, Z. Zhang, Dr. W. Liu, Prof. B. Wu, Prof. Y. Tao, Prof. Y. Chen
State Key Laboratory of Microbial Resources & CAS Key Laboratory of Microbial Physiological and Metabolic Engineering
Institute of Microbiology
Chinese Academy of Sciences
Beijing 100101, P. R. China
E-mail: chenyihua@im.ac.cn
Y. Ding, X. Li, M. Wang, Z. Zhang, B. Wu, Y. Tao, Y. Chen
University of Chinese Academy of Sciences
Beijing, China
Prof. G. P. Horsman
Department of Chemistry and Biochemistry
Wilfrid Laurier University
Waterloo, ON, N2L3C5, Canada

This PDF file includes:

Figures S1 to S5

Tables S1 to S3

SI References

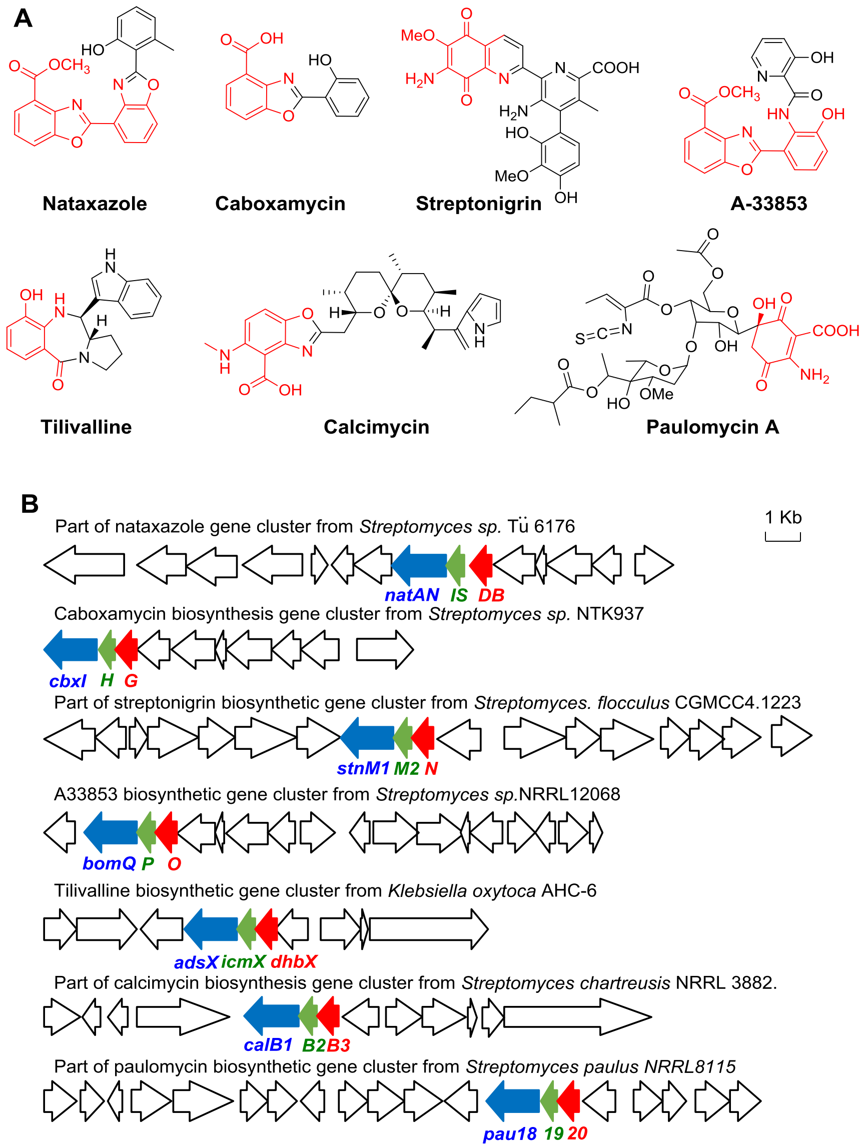

Figure S1. Natural products with 3-HAA derived substructures and their biosynthetic gene clusters. The three genes encoding ADIC synthase (blue), DHHA synthase (green), and DHHA dehydrogenase (red) are labeled in each cluster. DHHA dehydrogenases were proposed in these biosynthetic gene clusters but were not characterized biochemically until this study.

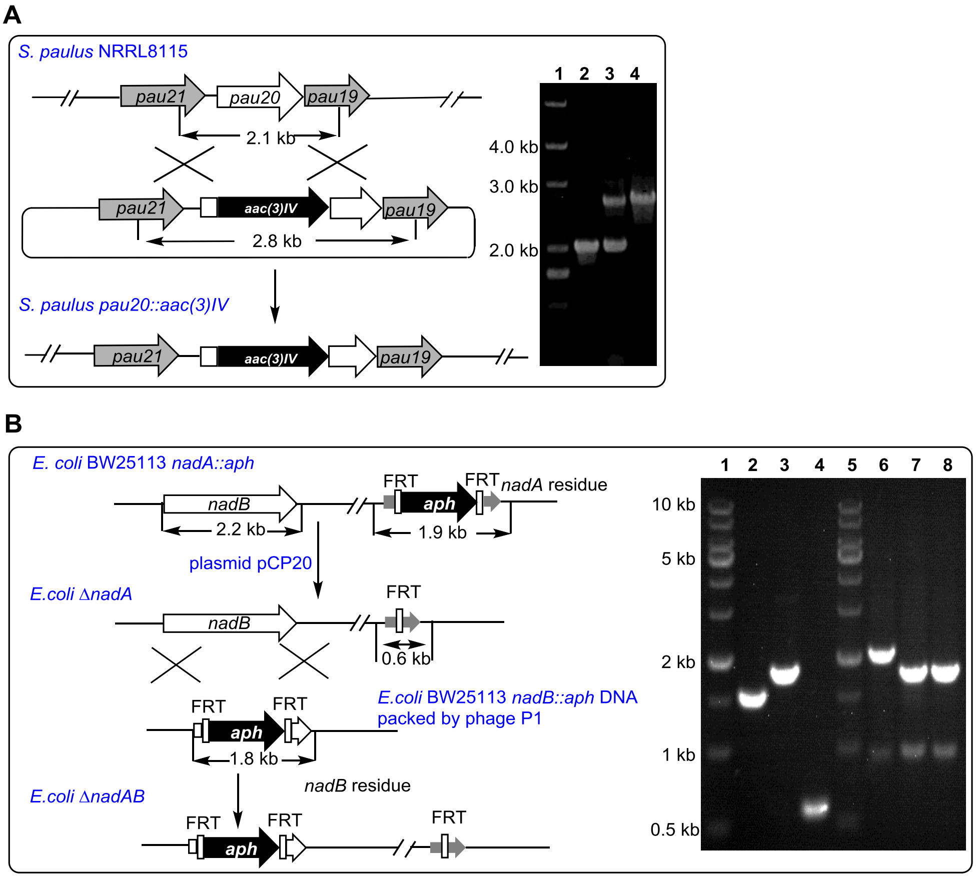

Figure S2. Construction of *S. paulus pau20::aac(3)IV* and *E. coli* Δ*nadAB*. (A) Illustration and PCR verification of *S. paulus pau20::aac(3)IV* construction: Lane 1, DNA marker; lane 2, *S. paulus* NRRL8115; lane 3, single crossover mutant; lane 4, *S. paulus pau20::aac(3)IV*. (B) Illustration and PCR verification of *E. coli* Δ*nadAB* construction: Lane 1 and 5, DNA marker; lane 2 and 6, *E. coli* BW25113; lane 3 and 7, *E. coli* BW25113 *nadA::aph*, lane 4 and 8, *E. coli* Δ*nadAB*. For lane 2-4 and lane 6-8, the PCR reactions were performed with primers nadA-F/nadA-R and nadB-F/nadB-R, respectively.

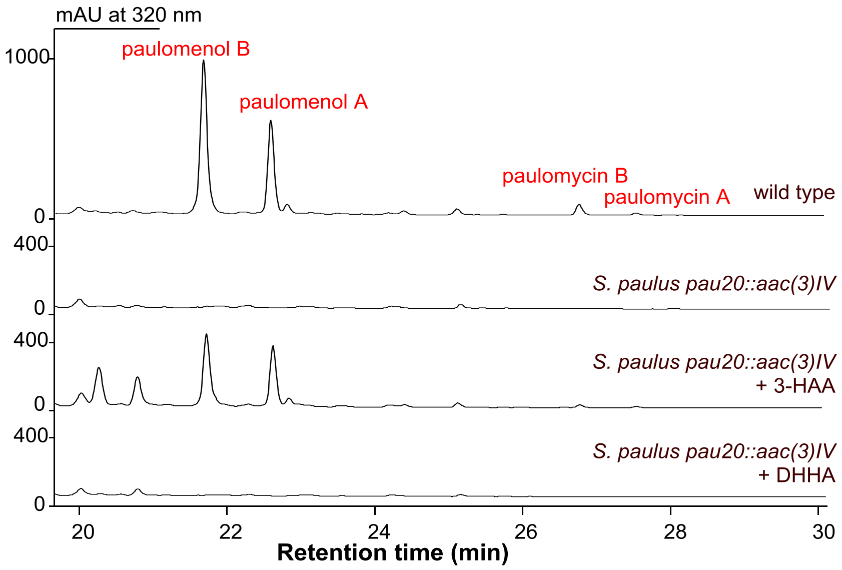

Figure S3. Characterization of Pau20 as a DHHA dehydrogenase *in vivo*. HPLC metabolite profiles of *S. paulus* wild type, the *pau20* inactivated mutant *S. paulus pau20::aac(3)IV*, and *S. paulus pau20::aac(3)IV* complemented with 3-HAA or DHHA.

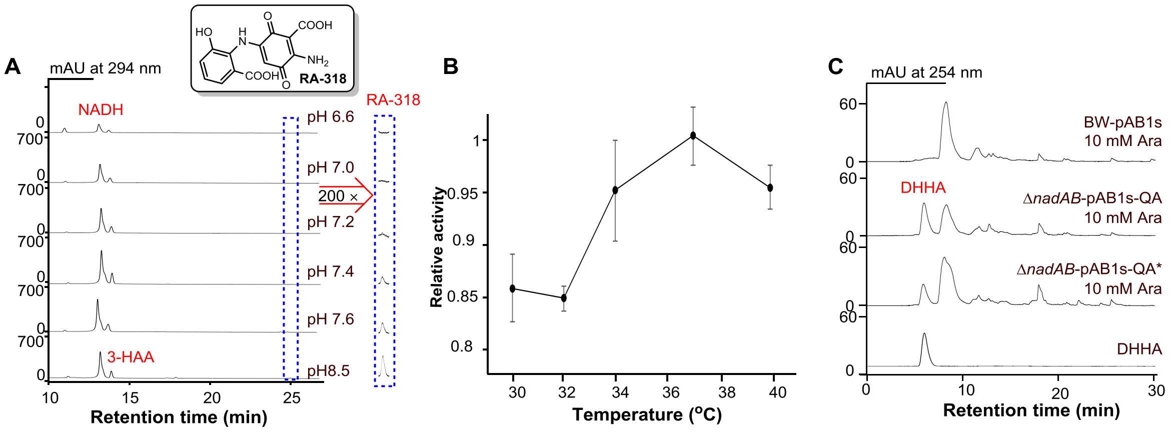

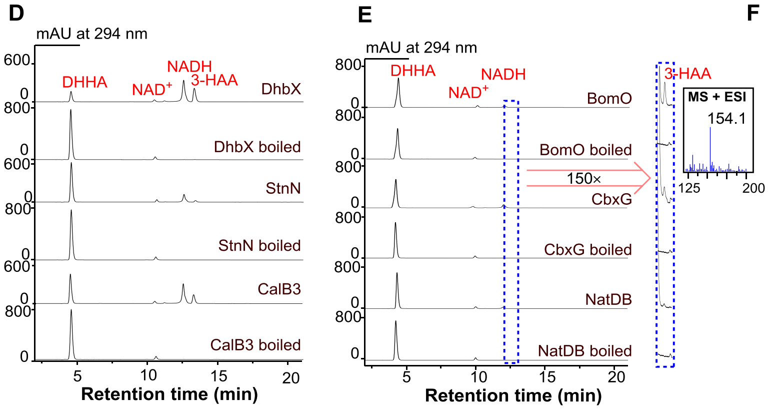

F

Kinetic parameters of DHHA dehydrogenases

Toward NAD+	K_m [mM]	k_{cat} [min^{-1}]	k_{cat}/ K_m [min^{-1} mM^{-1}]
Pau20	0.40 ± 0.025	25.42 ± 0.72	63.55 ± 2.17
CalB3	0.088 ± 0.017	11.06 ± 0.27	125.68 ± 21.21
DhbX	0.18 ± 0.054	50.44 ± 4.2	280.22 ± 60.73
StnN	0.33 ± 0.017	8.74 ± 0.14	26.48 ± 0.94

Toward DHHA	K_m [mM]	k_{cat} [min^{-1}]	k_{cat}/ K_m [min^{-1} mM^{-1}]
Pau20	5.18 ± 0.55	33.00 ± 2.26	6.37 ± 0.09
CalB3	2.96 ± 0.41	13.68 ± 1.10	4.62 ± 0.27
DhbX	1.69 ± 0.33	96.28 ± 11.08	56.97 ± 4.57
StnN	2.30 ± 0.31	12.84 ± 1.18	5.58 ± 0.24

Figure S4. Enzymatic studies to identify more efficient DHHA dehydrogenases. (A) Pau20 reactions in buffers with varied pH values: Pau20 assays were carried out at 37 °C for 30 min in buffers with pH values ranging from 6.6 to 8.5 unless specified. As previously reported,[1] compound RA-318 was the main spontaneously oxidized product of 3-HAA under the assay conditions; its structure was assigned by MS and NMR analyses (data not shown). (B) Optimization of Pau20 reaction temperature: Pau20 assays were carried out in 200 mM phosphate buffer (pH 7.0) for 30 min with temperatures ranging from 30 to 40 °C. (C) HPLC metabolite profiles of *E. coli* BW-pAB1s, Δ*nadAB*-pAB1s-QA and Δ*nadAB*-pAB1s-QA* fermentation broth to check the accumulations of DHHA in those strains during early stationary phase. (D) Representative assays of DHHA dehydrogenases DhbX, StnN, and CalB3. Those assays were carried out at 37 °C for 2 hours in 200 mM phosphate buffer (pH 7.0). (E) Representative assays of DHHA dehydrogenases BomO, CbxG, and NatDB. The production of 3-HAA was confirmed by LC-MS. Those assays were carried out at 37 °C for 2

hours in 200 mM phosphate buffer (pH 7.0). (F) Steady-state kinetic parameters of DHHA dehydrogenases Pau20, DhbX, StnN, and CalB3 at 37 ^{o}C pH 7.0. Data presented as mean ± SD, *n* = 3.

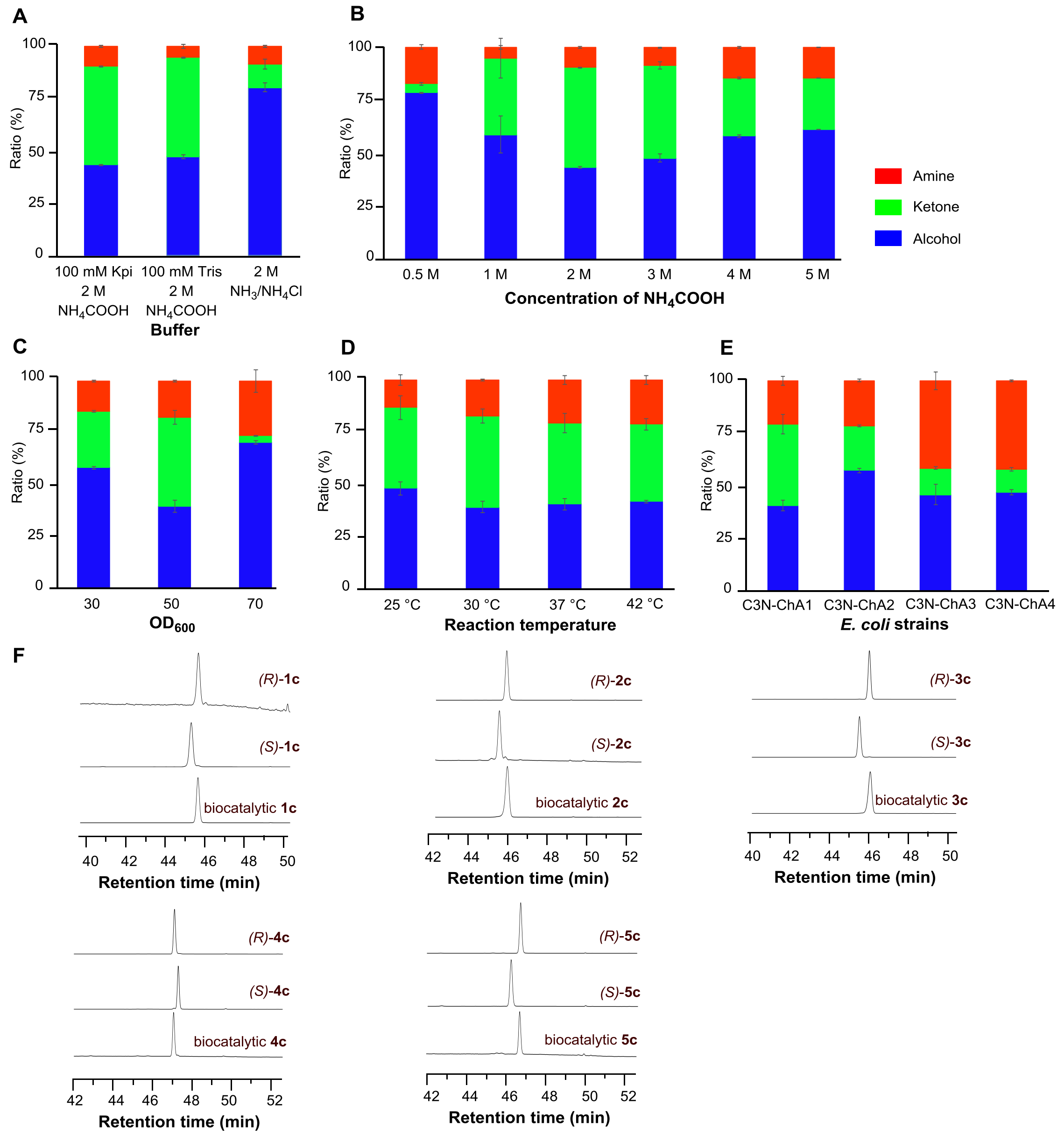

Figure S5. Optimization of the bioamination procedure using *rac*-1a as a substrate and enantiomeric purity analysis of the chiral amines produced by the C3N pathway-based whole-cell systems. (A) Optimization of catalytic buffers (pH 8.5): 5 mM *rac*-1a, OD_{600} = 30, under 30 °C, 230 rpm for 10 h in 1 mL reaction volume. All tested buffers contained 10% DMSO. (B) Optimization of the concentration of NH_4COOH: 100 mM KPi buffer (pH 8.5) with 10% DMSO, 5 mM *rac*-1a, OD_{600} = 30, under 30 °C, 230 rpm for 10 h in 1 mL reaction volume. (C) Optimization of biomass (OD_{600}): 100 mM KPi buffer (pH 8.5) with 10% DMSO, 5 mM *rac*-1a, 4 M NH_4COOH, under 30 °C, 230 rpm for 10 h in 1 mL reaction volume. (D)

Optimization of reaction temperature: 100 mM KPi buffer (pH 8.5) with 10% DMSO, 5 mM *rac*-1a, 4 M NH_4COOH, OD_{600} = 50, 230 rpm for 10 h in 1 mL reaction volume. (E) Detection of the bioamination capacities of different engineering strains: 100 mM KPi buffer (pH 8.5) with 10% DMSO, 5 mM *rac*-1a, 4 M NH_4COOH, OD_{600} = 50, under 37 ^{o}C, 230 rpm for 10 h in 1 mL reaction volume. (F) HPLC analysis of the enantiomeric purities of the chiral amines derivatized by FDAA (Marfey's Reagent). Data presented as mean ± SD, n = 3.

Table S1. The intracellular NAD(H) concentration of *E. coli*.[a]

**Strains**	NADH [mM]	NAD(H) [mM][b]
**Cultured in 3 mL medium[c]**		
E. coli BW25113[d]	0.16 ± 0.04	0.89 ± 0.04
E. coli BW25113	0.16 ± 0.03	0.85 ± 0.05
E. coli BW-pXB1s	0.13 ± 0.03	0.66 ± 0.05
E. coli BW-pAB1s	0.14 ± 0.04	0.68 ± 0.04
Δ*nadAB*-pXB1s-QA	0.31 ± 0.12	1.18 ± 0.18
Δ*nadAB*-pAB1s-QA	0.60 ± 0.16	4.43 ± 0.14
Δ*nadAB*-pAB1s-QA*	0.30 ± 0.09	7.21 ± 0.53
**Cultured in 50 mL medium[c]**		
E. coli BW25113	0.24 ± 0.01	0.96 ± 0.13
E. coli BW-pAB1s	0.28 ± 0.02	0.65 ± 0.09
Δ*nadAB*-pAB1s-QA*	0.31 ± 0.03	9.28 ± 0.76
**DMP cell factory[e]**		
E. coli C3N-DMP	1.53 ± 0.02	5.48 ± 0.03
E. coli DMP-Con	0.80 ± 0.05	2.05 ± 0.07
**chiral amine cell factory[e]**		
E. coli ChA1-Con	0.21 ± 0.04	1.52 ± 0.03
E. coli C3N-ChA1	0.32 ± 0.06	4.92 ± 0.50
E. coli C3N-ChA2	0.11 ± 0.03	5.37 ± 0.40
E. coli C3N-ChA3	0.21 ± 0.05	7.03 ± 1.57
E. coli ChA3-Con	0.25 ± 0.04	3.60 ± 0.47
E. coli C3N-ChA4	0.34 ± 0.20	6.38 ± 2.25

[a]: Data presented as mean ± SD, *n* = 3.;

[b]: NAD(H) means NAD^+ and NADH total concentration;

[c]: cultured in M9 medium with 10 mM arabinose and appropriate antibiotics unless noted specifically;

[d]: cultured in M9 medium without arabinose;

[e]: cultured in M9Y medium with 10 mM arabinose and appropriate antibiotics.

Table S2. Bacterial strains and plasmids.

**Strains or plasmids**	**Characteristics**[a]	**Reference or source**
Escherichia coli		
JM 109	General cloning host	Lab stock
BL21(DE3)	Host for protein expression	Novagen
ET12567/pUZ8002	Strain for intergeneric conjugation	Invitrogen
BW25113	Wild-type strain	Lab stock
BW25113 *nadA::aph*	BW25113 *nadA* mutant	ref. 2
BW25113 *nadB::aph*	BW25113 *nadB* mutant	ref. 2
BW25113 Δ*nadAB*	BW25113 *nadA & nadB* combined mutant	This work
BW-pXB1s-HAA	BW25113 harboring pXB1s-HAA	This work
BW-pXB1s	BW25113 harboring pXB1s	This work
BW-pAB1s	BW25113 harboring pAB1s	This work
Δ*nadAB*-pXB1s-QA	BW25113 Δ*nadAB* harboring pXB1s-QA	This work
Δ*nadAB*-pAB1s-QA	BW25113 Δ*nadAB* harboring pAB1s-QA	This work
Δ*nadAB*-pXB1s-QA*	BW25113 Δ*nadAB* harboring pXB1s-QA*	This work
Δ*nadAB*-pAB1s-QA*	BW25113 Δ*nadAB* harboring pAB1s-QA*	This work
BW25113(DE3)	Chassis cell for cell factory	Lab stock
DMP-Con	BW25113(DE3) harboring pAB1s and pRSF-*EcTdh-SpaNox*	This work
C3N-DMP	BW25113(DE3) harboring pAB1s-QA* and pRSF-*EcTdh-SpaNox*	This work
BW-ChA1-Con	BW25113(DE3) harboring pAB1s and pCDF-*TesADH-CalAmDH*	This work
BW-C3N-ChA1	BW25113(DE3) harboring pAB1s-QA* and pCDF-*TesADH-CalAmDH*	This work
BW-C3N-ChA2	BW-C3N-ChA1 harboring pACYC-*CalAmDH*	This work
BW-C3N-ChA3	BW25113(DE3) harboring pAB1s-QA* and pRSF-*TesADH-CalAmDH*	This work
BW-C3N-ChA4	BW-C3N-ChA3 harboring pACYC-*CalAmDH*	This work
BW-ChA3-Con	BW25113(DE3) harboring pAB1s and pRSF-*TesADH-CalAmDH*	This work
Streptomyces		
S. paulus NRRL 8115	Wild-type strain	NRRL
S. paulus NRRL 8115 *pau20::aac(3)IV*	*S. paulus* NRRL 8115 *pau20* mutant	This work
**Plasmids**		
pET28a	protein production vector, Kan^r	Novagen
pET28a-*pau20*	pET28a harboring *pau20*, Kan^r	This work
pET28a-*dhbX*	pET28a harboring *dhbX*, Kan^r	This work
pET28a-*calB3*	pET28a harboring *calB3*, Kan^r	This work
pET28a-*cbxG*	pET28a harboring *cbxG*, Kan^r	This work
pET28a-*stnN*	pET28a harboring *stnN*, Kan^r	This work
pET28a-*bomO*	pET28a harboring *bomO*, Kan^r	This work
pET28a-*natDB*	pET28a harboring *natDB*, Kan^r	This work
pAB1s (high-copy-number)	ColE1 origin, *ara*BAD promoter, Sm^r	ref. 3
pXB1s (medium-copy-number)	p15A origin, *ara*BAD promoter, Sm^r	ref. 3

pAB1s-HAA	pAB1s harboring *pau20*, *phzD* and *phzE*	This work
pXB1s-HAA	pXB1s harboring *pau20*, *phzD* and *phzE*	This work
pAB1s-QA	pAB1s harboring *nbaC*, *pau20*, *phzD* and *phzE*	This work
pXB1s-QA	pXB1s harboring *nbaC*, *pau20*, *phzD* and *phzE*	This work
pAB1s-QA*	pAB1s harboring *nbaC*, *dhbX*, *phzD* and *phzE*	This work
pXB1s-QA*	pXB1s harboring *nbaC*, *dhbX*, *phzD* and *phzE*	This work
pACYCduet-1	p15 origin, T7 promoter, Cm^r	Novagen
pCDFduet-1a	CDF origin, T7 promoter, Amp^r	Novagen
pRSFduet-1	RSF origin, T7 promoter, Kan^r	Novagen
pRSF-*EcTdh*-*SpaNox*	pRSFduet-1 harboring *EcTdh* and *SpaNox*	This work
pET28a-*TesADH*	pET28a harboring *TesADH*, Kan^r	This work
pET28a-*CalAmDH*	pET28a harboring *CalAmDH*, Kan^r	This work
pACYC-*CalAmDH*	pACYCduet-1 carrying *CaLAmDH*, Cm^r	This work
pCDF-*TesADH*	pCDFduet-1a harboring *TesADH*, Amp^r	This work
pCDF-*TesADH*-*CalAmDH*	pCDFduet-1a harboring *TesADH* and *CaLAmDH*, Amp^r	This work
pRSF-*TesADH*-*CalAmDH*	pRSFduet-1a harboring *TesADH* and *CaLAmDH*, Amp^r	This work

[a]: Kan^r, kanamycin resistance; Sm^r, streptomycin resistance; Amp^r, ampicillin resistance; Cm^r, chlorampenicol resistance.

Table S3. Oligonucleotides used in this work.

Name	Sequence(5′→3′)
pau20-s2	cagtcgattggctgacaattgattccgctcggcaggttcg
pau18R	cttgctagcagatgtcaattgatccgggccatcatcttcagt
pau22-R	taaaacgacggccagtgaattccatatggcgtcgcgccaccggcc
pau20-R	atcccttaacgtgagcctaggcagtcgtcctcggtgagttccag
pauN10ES	aaaactgcagcatatgggcacagccaattccgac
pauN10ER	cgggatccctggatgggcgtgagcgtc
pHAApau20-F	caggaggaattaaccatgggcacagccaattccg
pHAApau20-R	ctcctctttctctagacatatgctagcggcccagggtcgcgc
pHAAphzDE-F	catatgtctagagaaagagg
pHAAphzDE-R	accgagctcaccgaattcggatccttatgggcgacg
NbaC-F	gctaacaggaggaattaaccatgatgtttacctttggtaaac
NbaC-R	tcggaattggctgtgcccatctagtatttctcctctttctctagaggatccttacggctgatcac
nadA-F	tcaggcatcctcaatttc
nadA-R	ggcatacagctgaatctg
nadB-F	aacatcgcattatctgtg
nadB-R	gcgtagtgctgccagagc
T7	taatacgactcactatagg
Cal-P23R	gatgtaggtgttccacaggcaaaaaacccctcaagacccg
CALRS-F	aagaaggagatatacatatgtctaccgtgacctttg
CALRS-R	taccagactcgagggtacttagcgacgaacgcgccat
Cal-his-R	agtgcggccgcaagcttgtcgacttagcgacgaacgcgccatt

References

[1] M. K. Manthey, S. G. Pyne, R. J. Truscott, *Biochim. Biophys. Acta*. **1990**, *1034*, 207.

[2] T. Baba, T. Ara, M. Hasegawa,Y. Takai, Y. Okumura, M. Baba, K. A. Datsenko, M. Tomita, B. L. Wanner, H. Mori, *Mol. Syst. Biol*. **2006**, *2,* 2006.0008.

[3] Q. Cui, F. Zhou, W. Liu, Y. Tao, *Biotechnol. Lett.* **2017**, *39*, 1567.
